# A modified technique of two-staged extensor tendon reconstruction in zones 6–8 in a patient with absent palmaris/plantaris tendons: A case report

**DOI:** 10.1016/j.ijscr.2019.01.023

**Published:** 2019-01-30

**Authors:** Mohammad M. Al-Qattan, Saad A. Al Mohrij

**Affiliations:** a King Saud University, Riyadh, Saudi Arabia; b National Guard Health Affairs, Riyadh, Saudi Arabia; c Department of Surgery, College of Medicine, King Saud Bin Abdulaziz University for Health Sciences (KSAU-HS), Riyadh, Saudi Arabia

**Keywords:** Technique, Two-staged, Extensor tendon, Reconstruction

## Abstract

**Introduction:**

The indications for two-staged extensor tendon reconstruction are rare and only 14 previously reported cases were found in the literature. In these cases, silicone rods are inserted in the first stage. Few months later, the palmaris longus / plantaris tendon grafts are usually used to replace the silicone rods.

**Case report:**

we encountered a patient with major defects of the extensor tendons of all fingers extending from the proximal one third of zone 6 to zone 8. The patient had no palmaris or plantaris tendons. We utilized a modified technique of reconstruction using the split flexor carpi radialis as the tendon graft and the flexor carpi ulnaris as the motor tendon. At final follow-up, there was full active extension of the fingers. However, there was limitation of wrist flexion because of the harvesting of both wrist flexors.

**Discussion:**

We describe a modified technique of two-staged extensor tendon reconstruction which may be used in patients with absent palmaris/ plantaris tendons.

**Conclusion:**

In patients with absent palmaris/ plantaris tendons and major defects of the extensor tendons of all fingers, the use of split flexor carpi radialis is an adequate alternative for reconstruction and gives a good functional outcome.

## Highlights

• We encountered a patient with major defects of the extensor tendons.
• The patient had no palmaris or plantaris tendons.
• A modified technique of two-staged extensor tendon reconstruction was used.
• We utilized the split flexor carpi radialis as the tendon graft.
• At final follow up, there was full active extension of the fingers.

## 1 Introduction

Neglected flexor tendon injuries and failed flexor tendon repairs in zone 2 are frequently encountered in clinical practice; and management is done using two-staged flexor tendon reconstruction [1,2]. In contrast, two-staged extensor tendon reconstruction is a rare entity and is mainly indicated for major defects of the extensor tendons in zone 6; and requires the presence of supple metacarpophalangeal joints and the presence of adequate soft tissue coverage at the injury site. A review of the literature showed only 14 reported cases of two-staged extensor tendon reconstruction [[3], [4], [5], [6], [7]]. In the first stage a silicone rod is inserted; and the rod is then replaced by palmaris longus/ plantaris tendon graft in the second stage.

The authors encountered a patient with major defects of the extensor tendons of all fingers extending from the proximal one third of zone 6 to zone 8. The patient had no palmaris or plantaris tendons. We utilized a modified technique of reconstruction using the “split” flexor carpi radialis as the tendon graft. The work has been reported in line with SCARE Criteria [8].

## 2 Case presentation/description of the technique

**Fig. 1:**
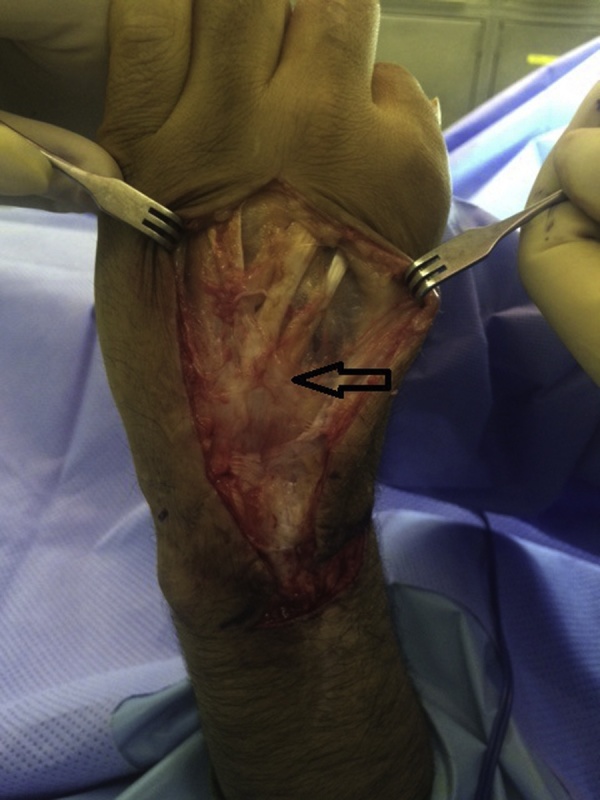
Exploration showing the extensor tendon defects in zone 6–8. Note that the extensor tendons are preserved in the distal two thirds of Zone 6 and then all tendons fade into a scar in the proximal part of Zone 6 (arrow). Also note the relatively preserved skin at the zone of injury because of the nature of original injury (skin degloving without skin loss).

**Fig. 2:**
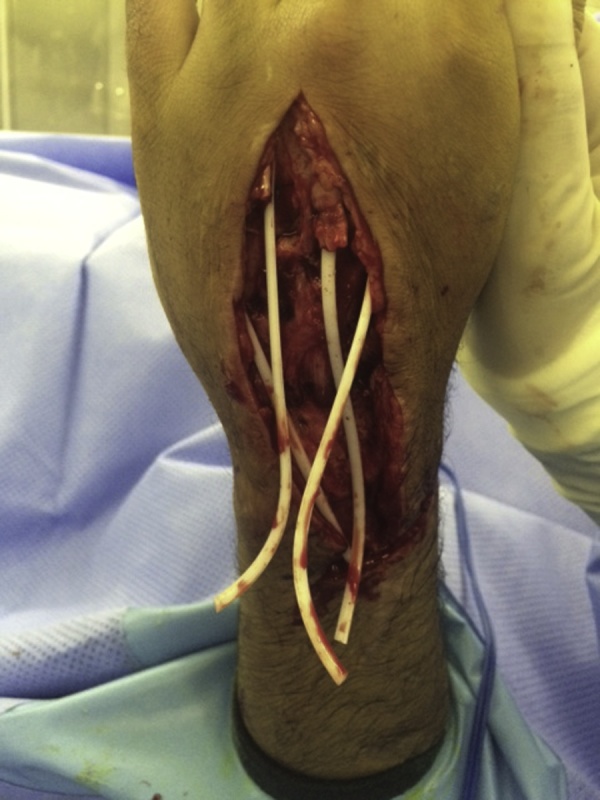
insertion of the four silicone rods. Note that the rods are sutured distally; but are left free (un-sutured) proximally.

**Fig. 3:**
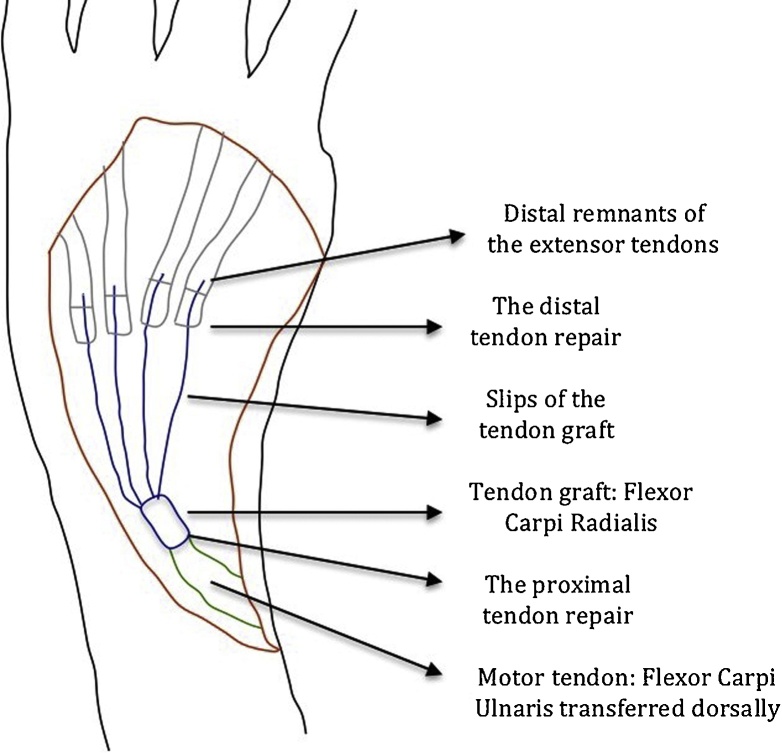
Diagrammatic demonstration of the second stage. The motor tendon is the transferred flexor carpi ulnaris. The tendon graft is the flexor carpi radialis tendon which is split distally to 4 slips (one slip to each finger). The proximal tendon repair is done first. Adjustment of tension is done during the distal tendon repair between the slips of the tendon graft and the remnants of the extensor tendons.

**Fig. 4:**
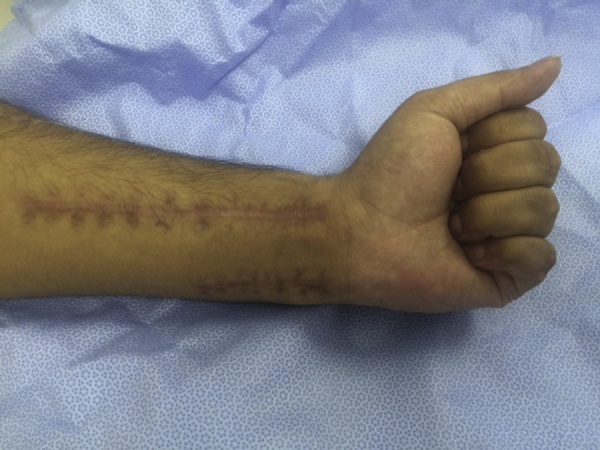
functional outcome at 1 year: Full active wrist extension/ finger flexion was maintained (as pre-operatively). Note the scars in the forearm from the tendon transfer and the harvesting of the tendon graft.

**Fig. 5:**
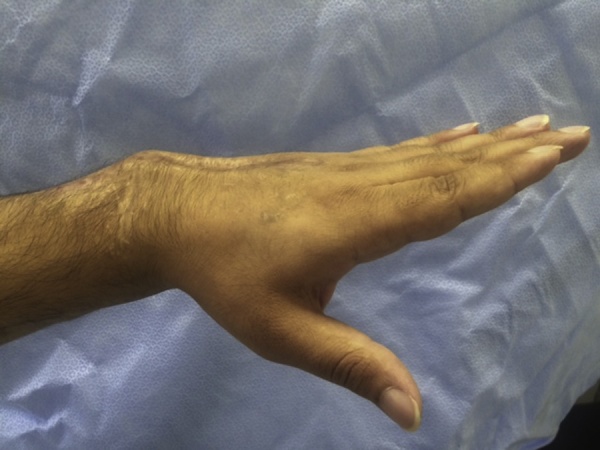
functional outcome at 1 year: Full active finger extension with simultaneous mild wrist flexion.

**Fig. 6:**
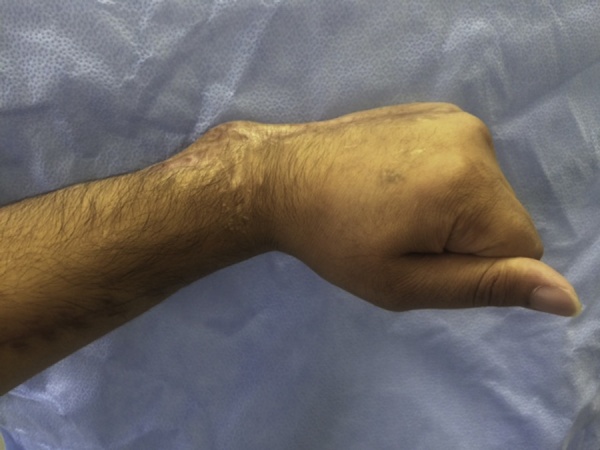
the only deficit noted at 1 year: Limitation of active wrist flexion attributed to harvesting of both wrist flexors.

A 30-year-old male presented to our clinic with inability to extend all fingers at the metacarpophalangeal joints of the left hand. One year prior to his presentation, he was involved in a car accident and was treated at a local hospital. The medical report indicated that the injury involved the dorsal aspect of the hand, wrist and forearm. There was degloving of the skin without skin loss. However, there was extensor tendon loss of all fingers extending from the proximal one third of zone 6 to zone 8 (including the musculotendinous junctions). Other than debridement, nothing was done to the extensor tendons; and the skin was closed primarily. On examination, there was no active extension at the metacarpophalangeal joints. There was no stiffness with full passive extension at the metacarpophalangeal joints. There were no deficits in active wrist extension/ finger flexion. The overlying skin was scarred but was thought to be adequate for soft tissue coverage. Two-staged extensor tendon reconstruction was planned. However, clinical examination showed the absence of palmaris longus tendon bilaterally. Ultrasound examination also showed the absence of plantaris tendon bilaterally. The patient was counselled regarding choices of the other sources of tendon grafts including: multiple toe extensors, “split” tensor fascia lata, and “split” flexor carpi radialis. The latter option was chosen and surgery was planned. In the first stage, exploration confirmed the presence of extensor tendon defects from the proximal one third of zone 6 to zone 8; including the musculotendinous junctions (Fig. 1). Four silicone rods were inserted. The rods were sutured distally to the remnants of the extensor tendons at the dorsum of the hand. All 4 rods were left free (un-sutured) in the distal forearm (Fig. 2). the patient resumed passive exercises of the metacarpophalangeal joints post-operatively to prevent stiffness. The second stage was done five months later (Fig. 3). The flexor carpi ulnaris tendon was cut near its insertion and was then transferred to the dorso-ulnar aspect of the forearm. The tendon of flexor carpi radialis was harvested as a tendon graft. The tendon graft was split distally in 4 slips on the operating table. The proximal tendon repair was done first between the transferred flexor carpi ulnaris tendon and the tendon graft using multiple figure-of-eight 3/0 polypropylene sutures. The splits of the flexor carpi radialis tendon graft were sutured to the proximal ends of the silicone rods. Each rod was then pulled out from the distal end; thereby introducing each slip into its corresponding pseudo-sheath induced by the rod. Each slip was then weaved through the corresponding remnant of the extensor tendon; and suturing was done using 4/0 polypropylene sutures (Fig. 3). In order to adjust tension of the repair, the wrist was positioned in 30° of extension. Tightening of the distal tendon repair was then done so that the metacarpophalangeal joints were 20° short of the full extension. A plaster cast was applied to the hand and the wrist to protect the repair. The cast was removed at 5 weeks and physiotherapy exercises were started. There were no post-operative complications. At final follow up (1 year later), full active wrist extension/ finger flexion was maintained (Fig. 4). Full active extension of the fingers at the metacarpophalangeal joints was demonstrated with slight wrist flexion (Fig. 5). As expected, active wrist flexion was limited to 20° because of the harvesting of the both wrist flexors (Fig. 6). Power grip was 83% of the contra-lateral hand. The patient was satisfied with the result and resumed his original job as a manual worker without any reported difficulties.

## 3 Discussion

From the clinical point of view, traumatic extensor tendon defects over zones 6–8 are usually seen with concurrent skin loss. In these cases, the best option of reconstruction would be a microsurgical free flap that contains vascularized tendons [9,10]. Free flap options include the radial forearm flap (which includes the palmaris longus or the flexor carpi radialis tendons), the dorsalis pedis flap (which includes the toe extensors), and thigh flaps (which include the fascia lata) [9,10]. In contrast, major defects of the extensor tendons over zones 6–8 in the presence of good soft tissue coverage is a rare entity; explaining the few reported cases in the literature [[3], [4], [5], [6], [7]]. In these cases, the overlying skin/subcutaneous tissue is always scarred; and hence two-staged reconstruction is preferred. The silicone rods provide vascularized smooth pseudo-sheaths for gliding of the tendon grafts [11]. Following the insertion of silicone rods, a highly vascularized pseudo-sheath is known to form around each rod [11]. The pseudo-synovial sheath is made up of 3 layers: an intima, media, and adventitia [11]. The intima secrets a glycosaminoglycan substance. The media is made of collagen and provide structural and vascular support. The adventitia is a highly vascular structure composed of loose fibrous tissue [11]. Hence, the pseudo-sheath is an excellent gliding surface for tendon grafts.

Generally, the best motor tendon is the original proximal cut ends of the extensor tendons in the forearm [3,4,7]. However, if the tendon defects include the musculotendinous junctions; another motor tendon is chosen. Al-Qattan [6] used the flexor carpi radialis as the motor tendon. Tomanio and Plakseychuk [5] used the flexor superficialis tendons as the motor tendons. In our patient, we used the flexor carpi ulnaris as the motor tendon and the flexor carpi radialis as a split tendon graft. This option was chosen because the latter tendon is relatively larger in diameter and hence is more suitable for splitting.

The best choice of tendon grafts has always been the palmaris/ plantaris tendons [[3], [4], [5], [6], [7]]. In our case, these tendons were absent; and hence we used the split flexor carpi radialis tendon as the tendon graft. A systematic review on the prevalence of palmaris tendon agenesis revealed an overall pooled rate of 20% [12]. The meta-analysis also showed that the absence rate is significantly affected by the ethnic background [12]. There were significantly lower pooled rates of palmaris tendon agenesis in Africans (11.3%) and East Asians (4.5%) when compared to Arab Middle Eastern population (41.7%) [12]. Our patient was an Arab. It is important to know that there is no association between an absent palmaris longus tendon and an absent plantaris tendon [13]. However, if one tendon (a palmaris or a plantaris tendon) is missing, there is a 67% chance that the contralateral tendon would also be missing [14]. Finally, there is no evidence that the absence of a palmaris or a plantaris tendon is associated with other musculoskeletal defects such as the absence of the flexor digitorum superficialis of the little finger [15].

Several messages from our case are learned. Firstly, the splitting of a single flexor carpi radialis tendon into four slips is possible and adequate for the reconstruction. From the theoretical point of view, the splitting destroys the outer gliding surface of the tendon graft. Our case demonstrates that the split tendon grafts will still glide well if inserted within the pseudo-sheaths induced by the silicone rods. Finally, if our technique is chosen, the patient has to be informed pre-operatively that there will be limitation of active wrist flexion due to the harvesting of both wrist flexors.

## 4 Conclusion

In patients with absent palmaris/ plantaris tendons and major defects of the extensor tendons of all fingers, the use of split flexor carpi radialis is an adequate alternative for reconstruction and gives a good functional outcome.

## Conflict of interest

None.

## Funding

None.

## Ethical approval

The study was approved by the Research Committee at National (CARE) Hospital.

## Consent

Written informed consent was obtained from the patient for publication of this case report and accompanying images. A copy of the written consent is available for review by Editor-In-Chief of this journal on request.

## Authors contribution

Both authors contributed significantly and in agreement with the content of the manuscript. Both authors participated in data collection and in writing of the manuscript.

## Registration of research studies

Not relevant here.

## Guarantor

M M Al-Qattan.

## Provenance and peer review

Not commissioned, externally peer-reviewed.
